# Life-Threatening Retroperitoneal Hemorrhage Caused by Lower Rib Fractures: A Case Report on Successful Management With Transcatheter Arterial Embolization

**DOI:** 10.7759/cureus.78992

**Published:** 2025-02-14

**Authors:** Keisuke Suzuki, Daiki Hirose, Yoshitaka Tomita, Chihiro Mori, Manabu Eiraku, Mako Sakakibara, Kazuki Kikuchi, Tatsuya Sugimoto, Gen Inoue, Masaharu Yagi, Kenji Dohi

**Affiliations:** 1 Department of Emergency, Critical and Disaster Medicine, Showa University School of Medicine, Tokyo, JPN

**Keywords:** blunt chest trauma, interventional radiology, lower rib fractures, retroperitoneal hemorrhage, trauma management

## Abstract

Rib fractures are commonly associated with pulmonary complications; however, they may occasionally cause retroperitoneal hemorrhage, which is a rare but life-threatening condition. We report the case of a 77-year-old male individual who presented with profound hemorrhagic shock after a fall. Imaging revealed fractures of the right 11th and 12th ribs with associated retroperitoneal hemorrhage. Despite a negative focused assessment with sonography for trauma, contrast-enhanced computed tomography revealed active vascular extravasation, necessitating emergency transcatheter arterial embolization. Hemostasis was achieved using n-butyl-2-cyanoacrylate, resulting in rapid stabilization and recovery. This case underscores the importance of considering retroperitoneal hemorrhage in patients with lower rib fractures and shock, particularly when the focused assessment with sonography for trauma findings is negative. This study also highlights the role of contrast-enhanced computed tomography and transcatheter arterial embolization in diagnosis and management, demonstrating the need for a multidisciplinary approach to trauma care.

## Introduction

Rib fractures are common injuries from blunt chest trauma. Complications associated with rib fractures often include pulmonary issues such as pneumothorax and lung contusion. The incidence of these complications, along with mortality rates, tends to increase as the number of rib fractures increases [1,2]. Additionally, mortality and complication rates have been shown to increase in the elderly if rib plating is not performed; this means observation alone may not be sufficient in some cases [3]. Although vascular complications from rib fractures are less common than pulmonary complications, they can be life-threatening when they occur. Intercostal artery injuries and injuries to other thoracic vessels have been reported; however, retroperitoneal hemorrhage secondary to rib fractures is a rare and thus potentially overlooked complication [4]. In this report, we present a case in which a patient was transported to the emergency department with life-threatening shock because of retroperitoneal hemorrhage caused by rib fractures. Bleeding was successfully controlled using transcatheter arterial embolization (TAE).

## Case presentation

A 77-year-old male individual with a history of anticoagulant and antiplatelet therapy for atrial fibrillation and iliac artery stent presented to the emergency department after falling at home. He described a sudden onset of weakness and an inability to move following a fall. On arrival, the patient complained of severe back pain. His vital signs were unmeasurable blood pressure with a heart rate of 139 bpm, and he had elevated lactate levels of 13.3 mmol/L (Table 1), consistent with a state of profound shock.

**Table 1:** Laboratory data on arrival. WBC, white blood cell; Hb, hemoglobin; Plt, platelet; BUN, blood urea nitrogen; Cre, creatinine; AST, aspartate aminotransferase; ALT, alanine aminotransferase; CRP, C-reactive protein; PT-INR, prothrombin time-international normalized ratio; APTT, activated partial thromboplastin time.

Parameter	Test result	Reference range
WBC (/μL)	9,900	3,900-9,700
Hb (g/dL)	10.3	13.4-17.1
Plt (×10^4^/μL)	33.4	15.3-34.6
BUN (mg/dL)	21.3	9.0-21.0
Cre (mg/dL)	1.97	0.60-1.00
AST (U/L)	88	5-37
ALT (U/L)	27	6-44
CRP (mg/dL)	0.49	<0.30
PT-INR	1.05	0.90-1.10
APTT	25.6	24.0-34.0
D-dimer (μg/dL)	8.30	<1.00
Lactate (mmol/L)	13.3	0.6-1.4

**Figure 1:**
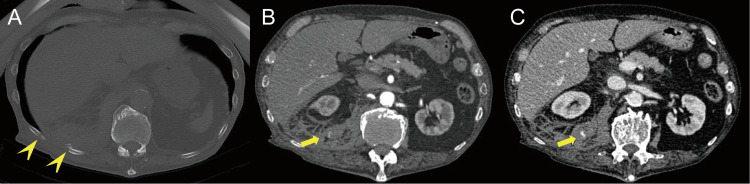
Pretreatment imaging. (A) Non-contrast CT scan with a bone window, (B) 30 seconds after injection of contrast medium, and (C) 80 seconds after injection of contrast medium. Axial non-contrast CT images show two fractures of the right 11th and 12th ribs (arrowhead). Axial contrast-enhanced CT showing a retroperitoneal hematoma with active extravasation (arrow).

Physical examination revealed peripheral coldness and tenderness in the thoracic spine and ribs but no apparent external bleeding. Focused assessment with sonography for trauma (FAST) was negative for intraperitoneal free fluid. The patient was promptly administered a rapid infusion of 1000 ml of extracellular fluid, and his vital signs stabilized (heart rate, 105 bpm; blood pressure, 117/63 mmHg). Thus, he was judged to be in responder shock and underwent a computed tomography (CT) scan. Contrast-enhanced CT of the torso revealed fractures of the right 11th and 12th ribs (Figure 1A), as well as retroperitoneal hemorrhage with extravasation of contrast, suggesting active vascular injury (Figures 1B, 1C).

**Figure 2:**
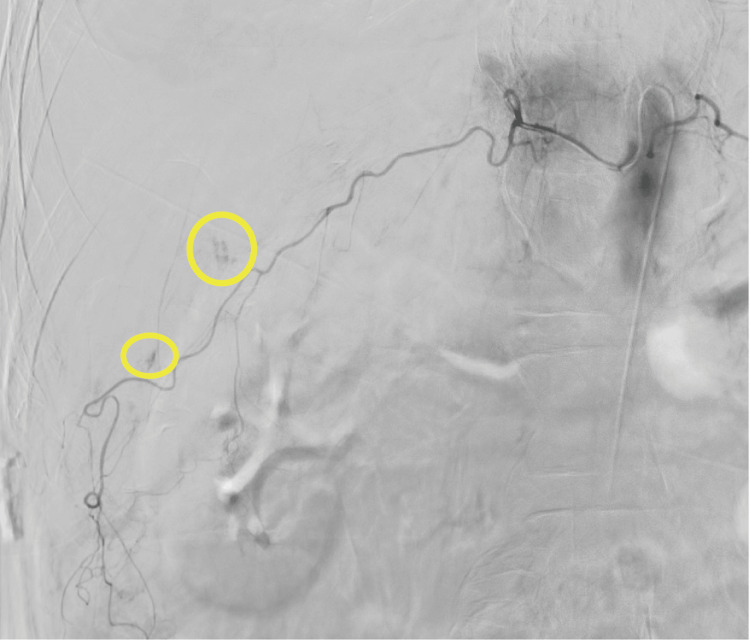
Angiography. Digital subtraction angiography revealed active bleeding from a right subcostal artery (circle).

Based on these findings, TAE was performed. Angiography revealed a right subcostal artery as the source of bleeding (Figure 2). Embolization was successfully performed using n-butyl-2-cyanoacrylate (NBCA), achieving hemostasis. After the procedure, the patient’s hemodynamic status improved substantially, with stabilization of the vital signs (heart rate, 75 bpm; blood pressure, 113/62 mmHg) and normalization of lactate levels (1.55 mmol/L). The patient was managed postoperatively in the intensive care unit where he received supportive care, including fluid resuscitation and blood transfusion. On the second day of hospitalization, the patient was transferred to a rehabilitation facility for continued recovery. At the time of transfer, he could mobilize with assistance.

## Discussion

Rib fractures caused by blunt trauma are frequently associated with lung injuries such as pneumothorax and pulmonary contusion, and clinical management often requires a comprehensive understanding of the potential complications. Although lower rib fractures can sometimes be accompanied by liver or splenic injuries, they can cause retroperitoneal hematomas in rare cases [4]. This is because some intercostal arteries on the dorsal side of the lower ribs run close to the retroperitoneal cavity.

In this case, the patient presented with life-threatening shock caused by a retroperitoneal hemorrhage secondary to fractures of the right 11th and 12th ribs. The absence of significant findings on the initial FAST underscores the diagnostic challenges associated with retroperitoneal bleeding. Unlike intraperitoneal hemorrhage, retroperitoneal hemorrhage is often unseen on ultrasound because of its deep anatomical location and complex surroundings. According to previous reports, retroperitoneal hematomas were detected in all cases where CT or MRI was performed; however, in 19 cases where ultrasound was performed, retroperitoneal hematomas were detected in only 12 cases [5]. Additionally, the time from injury to diagnosis is crucial in cases of retroperitoneal hemorrhage. Specifically, delayed diagnosis can lead to notable morbidity and mortality, particularly in older patients or those on anticoagulation therapy [6]. Thus, contrast-enhanced CT plays a pivotal role in identifying the source of bleeding and in guiding further management.

TAE was instrumental in achieving hemostasis. TAE is widely recognized as a minimally invasive and effective intervention for controlling arterial bleeding, particularly in patients who are hemodynamically unstable or are poor candidates for surgical intervention. It is known for its high efficacy in treating retroperitoneal hemorrhage [7]. The NBCA used in this case can be used for embolization without relying on coagulation ability. Thus, it is often useful for elderly patients who take oral anticoagulants. In this case, embolization of the right subcostal artery using NBCA not only stabilized the patient but also allowed for a rapid recovery trajectory, as evidenced by the patient’s transfer to a rehabilitation facility on the second hospital day. In particular, older patients are prone to complications from rib fractures; therefore, it is of great relevance that their condition quickly improves [8].

A limitation of this report is that it only covers the acute phase and does not include the long-term follow-up. However, this case highlights several important considerations for the management of patients with blunt trauma and rib fractures. First, if a trauma patient with shock is FAST-negative and a lower rib fracture is suspected, retroperitoneal hemorrhage due to a lower rib fracture should be considered as a differential. Second, prompt utilization of contrast-enhanced CT is essential for accurate diagnosis and localization of the bleeding source. Finally, interventional radiology services for TAE can be lifesaving in such scenarios, emphasizing the need for various approaches to trauma treatment, not just surgery.

## Conclusions

This case emphasizes the importance of understanding the anatomical and pathophysiological nuances associated with lower rib fractures. Although uncommon, retroperitoneal hemorrhage should be considered in the differential diagnosis, particularly when patients present with shock and negative FAST findings. Early recognition and timely intervention are critical for improving outcomes in rare but potentially fatal cases.
